# Multi Self-Healable UV Shielding Polyurethane/CeO_2_ Protective Coating: The Effect of Low-Molecular-Weight Polyols

**DOI:** 10.3390/polym12091947

**Published:** 2020-08-28

**Authors:** Mohammad Mizanur Rahman, Rami Suleiman, Md. Hasan Zahir, Aasif Helal, A. Madhan Kumar, Md. Bashirul Haq

**Affiliations:** 1 Center of Research Excellence in Corrosion, King Fahd University of Petroleum and Minerals, Dhahran 31261, Saudi Arabia; ramismob@kfupm.edu.sa (R.S.); madhankumar@kfupm.edu.sa (A.M.K.); 2 Center of Research Excellence in Renewable Energy, King Fahd University of Petroleum and Minerals, Dhahran 31261, Saudi Arabia; hzahir@kfupm.edu.sa; 3 Center of Research Excellence in Nanotechnology, King Fahd University of Petroleum and Minerals, Dhahran 31261, Saudi Arabia; aasifh@kfupm.edu.sa; 4 Department of Petroleum Engineering, College of Petroleum and Geosciences, King Fahd University of Petroleum and Minerals, Dhahran 31261, Saudi Arabia

**Keywords:** polyurethane, self-healing, coating

## Abstract

We prepared a series of polyurethane (PU) coatings with defined contents using poly(tetramethylene oxide)glycol (PTMG) with two different molecular weights (i.e., *M_n_* = 2000 and 650), as well as polydimethyl siloxane (PDMS) with a molecular weight of *M_n_* 550. For every coating, maximum adhesive strength and excellent self-healing character (three times) were found using 6.775 mol% mixed with low-molecular-weight-based polyols (PU-11-3-3). Defined 1.0 wt% CeO_2_ was also used for the PU-11-3-3 coating (i.e., PU-11-3-3-CeO_2_) to obtain UV shielding properties. Both the in situ polymerization and blending processes were separately applied during the preparation of the PU-11-3-3-CeO_2_ coating dispersion. The in situ polymerization-based coating (i.e., PU-11-3-3-CeO_2_-P) showed similar self-healing properties. The PU-11-3-3-CeO_2_-P coating also showed excellent UV shielding in real outdoor exposure conditions.

## 1. Introduction

An organic coating deposited on a substrate surface can enhance the protection of that surface, which can ultimately increase the lifespan of that substrate and its structures. These coatings can provide long-term performance, giving them priority in industry [1,2,3,4]. Coating longevity also depends on the geographical location and indoor or outdoor application conditions. Any coatings used in outdoor atmospheric conditions where sunlight, high temperatures, humidity, and marine aerosols are common need extended protective properties [2,4]. Otherwise, the coating might be damaged within a short amount of time. In such conditions, if there is any micro-damage in the coating, the damage is rapid due to the easy passage of electrolytes (i.e., marine aerosols and water vapor). Scientists have previously tried to obtain extended coating protective properties by using self-healing, crosslinking, and multifunctional techniques in organic coatings [4,5,6,7].

It is common for a coating to be scratched or damaged during its service life. In such conditions, the coating loses its protective property rapidly. To repair the scratch or damage, the coating should heal properly. To heal the coating, a common trend is to give the coating a self-healing property. Self-healing can be defined as the property by which a coating can heal from damage without external activity. There are different ways to make a coating self-healable. Both intrinsic and extrinsic processes are used in the coating industry. In the extrinsic process, additional self-healing agents are added during the coating dispersion preparation. The self-healing agent is released when there is dry coating damage in a real application. In the intrinsic process, no additional agent is used to confer the healing property. The coating obtains this property from hydrogen bonds, van der Waals forces, and the supramolecular structure [8,9,10,11,12]. 

There are several varieties of organic coatings used in outdoor atmospheric conditions. A common trend is to use multiple coatings to protect the structure. These can be classified as a primer, mid-coat, and top coat. Different epoxy compounds are mainly used as a primer and mid-coat [1,2,3]. Polyurethane (PU) is used mainly as a topcoat, especially when UV degradation is a concern. PU coating consists of urethane–urea groups, which come from polyol reactions with isocyanate/amine. Different polyether, polyester, hydroxyl-terminated siloxane, and hydroxyl-terminated acrylate are widely used as polyols [1,13,14,15]. The PU properties are tuned using different polyol contents and different molecular weights [13,15,16]. Usually, polyols with high molecular weight make the coating stiffer, more adhesive, and more corrosion resistant [17]. Polyols with low molecular weight make the coating less rigid [16,17]. To gain the target property, mixed polyols are considered in PU [13,16].

Polydimethyl siloxane (PDMS) is a popular polyol in PU coatings. It has been widely used to improve coating hydrophobicity, corrosion resistance, and antifouling resistance. However, the performance highly depends on the PDMS molecular weight and its contents. In fact, improved protective properties have been recorded using PDMS and other polyether or polyester polyols in PU [13,16,18].

A PU coating can easily be degraded in the presence of UV rays. To slow down UV degradation, mixing a variety of nanoparticles into the coating can improve UV degradation resistance. Different nanoparticles (i.e., metal oxides, carbon nanotubes, and clays) are widely used to enhance coating resistance against UV degradation. However, the effects of nanoparticle content are quite confusing, and only certain contents can improve this property. Saudi Arabia is geographically located in such conditions, where high temperature and high humidity are common during the summertime. Additionally, marine aerosols create harsh conditions in and around coastal areas. Thus, an early failure of organic coatings is common in Saudi Arabia. Rahman et al. found pristine PU coating degradation within a few months during open atmospheric conditions. They also used functionalized multiwalled carbon nanotubes (MWCNTs) in polyurethane (PU) to improve UV degradation resistance. However, UV degradation did not stop completely and was instead slowed down heavily when using 2.0 wt% carboxyl-functionalized MWCNTs. After 1 year, the PU coating with 2.0 wt% carboxyl-functionalized MWCNTs had slightly degraded. Recently, Rahman et al. showed improved UV degradation resistance for PU coatings by adding different metal oxides. The metal oxides act as a UV shielding, and hence, the UV degradation is reduced. The CeO_2_, ZnO, and TiO_2_ are widely being used as UV shielding materials in PU coatings [4,7,19]. There are other materials also used in UV shielding. Especially, different types of dye, carbon, and polydopamine are also used as UV-shielding materials [20,21,22,23,24,25,26]. However, the CeO_2_, similar to other metal oxides, is an attractive choice as it is significantly less toxic, cheap, and possesses filler properties [4,7].

Rahman et al. also explained different methods of adding metal oxides to PU coatings. Many factors affected homogeneous metal oxide mixing in the PU coating. The most widely used methods for homogeneous metal oxide dispersion include metal oxide functionalization, sonication during mixing, and proper metal oxide content. Mainly, blending and in situ polymerization are considered to make homogeneous metal-oxide-based PU dispersions [7].

UV degradation resistance and the self-healing of polymers has also been separately worked on [20,21,22,23,24,25,26,27,28,29,30,31]. Zhang et al. prepared polyethylenimine-based elastomers with self-healing property [27]. The elastomer restores 90% tensile strength of that elastomer. Peng et al. prepared a coating, based on a host–guest complex [28]. The coating showed excellent UV shielding and self-healing properties. Wang et al. prepared a gel that had the capacity to 100% self-heal [29]. Dong et al. prepared a biodegradable superhydrophobic polydopamine-based self-healing and UV-shielding coating [26]. Kim et al. prepared supramolecular PU, which showed 96% self-healing efficiency based on a tensile test [12]. Wang et al. also prepared siloxane-based PU that possessed the healing nature of mechanical damage [30]. However, it is hard to find any report on dual UV degradation resistance and the self-healing activity for a single PU coating. There are no existing reports on the effect of mixed polyols (low- and high-molecular-weight polyols) on the combined self-healing and UV degradation resistance of PU coatings. Self-healing highly depends on the chain entanglement of a polymer. The low-molecular-weight polyol chains are flexible, which may favor self-healing in the final polymer chain entanglement. However, the low-molecular-weight-based polyols make polymers less mechanically strong and more brittle. Our previous works confirmed that mixed polyols (a small content of low-molecular-weight-based polyols with high-molecular-weight-based polyols) improved hydrophobicity (only from PDMS), adhesive strength, and elasticity. Therefore, a mixed polyol with low-molecular-weight-based polyol might be a proper self-healing choice without sacrificing their protective properties. In current formulations, three polyols had been chosen to use the advantages of each polyol. The PTMG-2000 will maintain the mechanical strength, PTMG-650 can favor self-healing, and PDMS can improve both the hydrophobicity and self-healing. Low-molecular-weight-based polyols could not be used due to their very weak mechanical properties [16,17].

In this report, a series of PU dispersions (see Table 1) was synthesized using small-molecular-weight polyols (650 and 550) with the large-molecular-weight-based polyol poly(tetramethylene oxide)glycol (PTMG) *M_n_* = 2000. The dispersion was used to coat mild steel substrates. All coatings were considered for (1/2/3/4) self-healing. The basic coating properties, such as adhesive strength and hydrophobicity, were also evaluated. The promising coatings (PU-11-3-3) were examined for their UV degradation resistance. To enhance UV degradation resistance, a defined CeO_2_ content (1.0 wt%) was mixed with PU via either blending or in situ polymerization. These coatings were also considered for a self-healing test to evaluate the effect of CeO_2_ on self-healing properties. The best-performing self-healed coatings were further considered for their UV shielding in real outdoor atmospheric conditions.

## 2. Materials and Methods

### 2.1. Materials

Poly(tetramethylene oxide) glycol (*M_n_* = 650 and 2000), PDMS (*M_n_* = 550), triethylamine, *N*-methyl-2-pyrrolidone, 4,4-dicyclohexylmethane diisocyanate, ethylene diamine, dimethylolpropionic acid, methylethylketone (MEK), and dibutyltin dilaurate were all received from Sigma Aldrich, St Louis, Mo, USA. CeO_2_ nanoparticles were prepared in accordance with our previous report [31].

### 2.2. PU Dispersion Preparation

The basic polymerization steps were kept the same during the preparation of all dispersions based on previous reports. In all cases, the pre-polymer was prepared by charging PTMG, DMPA solution with NMP and H_12_MDI. The MEK (10.0 wt%) was added into the pre-polymer to reduce viscosity. Later, the pre-polymer was neutralized, dispersed, and chain extended by using the proper amount of TEA, H_2_O (70.0 wt%), and EDA (mixed with 10 ml water). The pre-polymer formation and reaction termination were monitored via FT-IR analysis.

### 2.3. PU–CeO_2_ Dispersion Preparation via In Situ Polymerization

All of the polymerization steps were kept exactly the same, except for the addition of CeO_2_. The CeO_2_ (1.0 wt%) was premixed with acetone (15 mL) and ultrasonicated for 30 min. The CeO_2_–acetone mixture was added into a premixed DMPA–NMP solution and stirred further for 30 min at 45 °C. This solution was added into a polyol and then charged with H_12_MDI.

### 2.4. PU-CeO_2_ Dispersion Preparation via a Blending Process

The CeO_2_ (1.0 wt%) was premixed with acetone (15 mL) and ultrasonicated for 30 min. Then, the CeO_2_–acetone mixture was added directly into a PU dispersion. The mixture was stirred for 1 h at 30 °C.

### 2.5. Substrate Coating

The coating on the substrate was fixed at 100 μm under wet conditions. The coating was made using a bar auto-coater. All prepared wet coatings were dried at ambient conditions for 48 h. To remove the used solvents completely, the earlier dried coatings were further dried for 12 h at 65–70 °C in an oven.

### 2.6. Self-Healing Test

The self-healing test was conducted at 50 °C. Each coating was cut by a sharp blade. To heal the coating, the tested coating was kept in an oven for a maximum of 12 h. The healing was checked via optical microscopy after every 1 h.

### 2.7. Characterization

Different techniques were applied to identify coating properties. In all cases, the value was the average of five testing results. The reaction monitoring was performed and the final polymer was identified via FT-IR (Impact 400D, Nicolet, Madison, WI, USA) analysis. The photographs of coatings were obtained with a Hitachi S-4200 (Tokyo, Japan) field emission scanning electron microscope (FESEM) at 5 kV. A Theta Optical tensiometer instrument (Attension, Helsinki, Finland) was used to obtain the water contact angle. The ASTM D4541 method was followed to evaluate adhesive strength using the pull-off test. XPS (ESCA 250 XPS, Thermo Scientific, East Grinstead, UK) analysis was done to characterize the surface of the coating. The ASTM D2240-75 specification was followed to evaluate the Shore A hardness test using the LX-A rubber Hardness Tester (Shanghai Liuling Instrument Company, Shanghai, China). An exposure test was used to check the UV shielding property of the coating and was based on our previous report [31].

## 3. Results and Discussion

**Figure 1:**
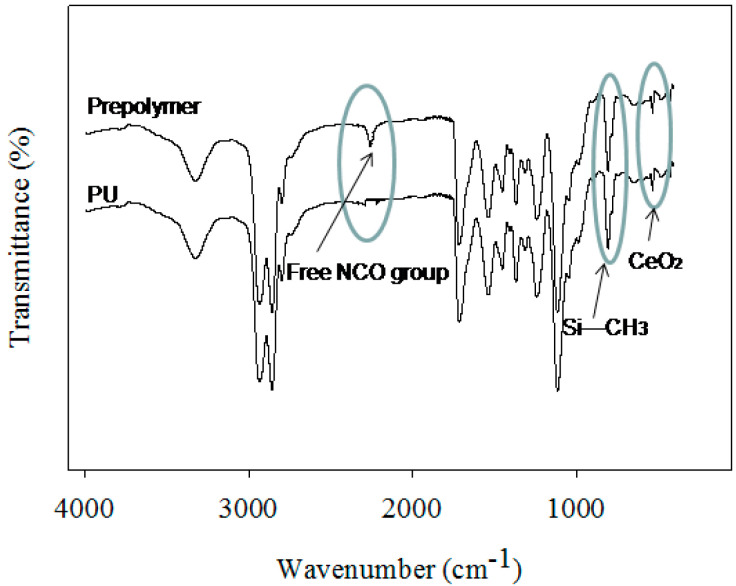
FT-IR spectra of PU-11-3-3-CeO_2_-P coating.

**Figure 2:**
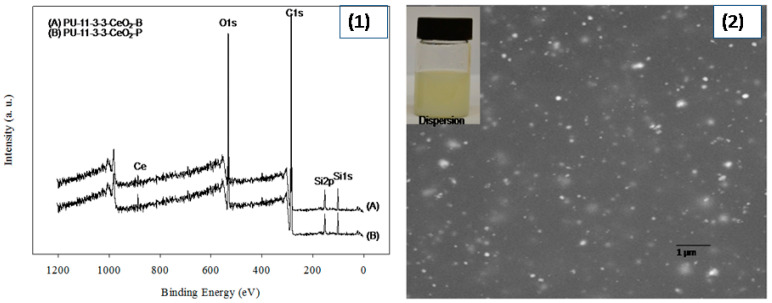
XPS spectra (**1**) and SEM (**2**) of PU-11-3-3-CeO_2_-P coating (insight dispersion).

A series of 17 formulated coatings were prepared based on different polyol contents (see Table 1). A defined CeO_2_ content was also used in PU-11 coatings. The different steps of PU preparation were monitored via FT-IR analysis (see Figure 1). The polyol, DMPA, and H_12_MDI formed the pre-polymer. Usually, the appearance and disappearance of a peak at 2170 cm^−1^ for the NCO group is considered a tool for monitoring the reaction [4]. During the pre-polymer formation stage, a strong peak appeared at 2170 cm^−1^ due to the unreacted NCO group. The peak remained during the neutralization and dispersion steps. During chain extension in the last stage, the EDA reacted with the NCO group and the peak disappeared within 1 h, confirming the completion of the reaction. All the prepared coatings showed identical PU functional groups, such as urethane (NHCOO) and urea (NHCOO), as the peaks appeared at 3400 cm^−1^ (for NH groups) and 1706 cm^−1^ (for CO groups). A peak at 816 cm^−1^ for the CH_3_ group attached with Si was also recorded for coatings using PDMS and thus confirmed the presence of PDMS in PU. Moreover, for PU–CeO_2_ coatings, a new peak appeared at 560 cm^−1^, which also confirmed the presence of CeO_2_ in the PU coatings. The peak at 560 cm^−1^ was similar for the coatings prepared via blending or polymerization. It was not possible to identify the interaction between the CeO_2_ and PU groups clearly, as the PU–CeO_2_ coatings were prepared via two processes. XPS analysis was used to evaluate the effect of the blending and polymerization preparation techniques on CeO_2_ and PU interactions (see Figure 2). As expected, peaks were recorded at 100, 285, 400 and 530 eV, which reflected Si, C, N and O, respectively. Two additional peaks around 887 and 889 eV were also recorded for samples containing CeO_2_. Both peaks had slightly higher values in the blended sample. Moreover, slightly higher-intensity peaks were recorded for coatings made via the polymerization method. Most importantly, a minor new peak also appeared at 897 eV for the blended coatings. This peak and the pristine CeO_2_ peak were almost at the same position. Thus, the CeO_2_ had comparatively less interaction with PU in the blended coating. On the other hand, the CeO_2_ particles entered the polymer chain by interacting with carboxyl groups in the polymerized coating. However, the CeO_2_ was homogeneously distributed in both PU–CeO_2_ coatings. Neither precipitation nor phase separation was seen in PU–CeO_2_ dispersions (see Figure 2). To confirm the CeO_2_ distribution in coatings, SEM analysis was done. As a small content of CeO_2_ was used, the different synthesis process had no noticeable effect on CeO_2_ distribution. CeO_2_ was homogeneously distributed mostly in both cases. A typical SEM photograph is shown in Figure 2.

Coating hardness is an important criterion. As hardness implies polymer chain stiffness, low hardness means polymer chain deformation and fast entanglements. The respective hardness values are summarized in Table 2. As the low-molecular-weight polyol content decreased, hardness also increased. Among all of the pristine PU coatings, the maximum hardness value was recorded for the PU-18 coating. The lower hardness values were recorded for the coatings that have a higher content of low molecular weight-based polyol. The PU-13 and PU-15 coatings had hardness values closer to that of the PU-18 coating. Only 2.25 and 4.525 mol% low-molecular-weight polyol replaced high-molecular-weight polyol, and the polymer deformation structure only slightly deviated in the PU-13 and PU-15 coatings. A moderately decreased value was recorded for the PU-11 coatings. The PU-CeO_2_ coatings showed higher values than their parent PU coating. The CeO_2_ filler interacted with the PU groups, and these polymers had greater stiffness, which reflected higher hardness. At the same time, the blended coatings’ hardness values were less than those of the polymerized coatings. This also implies that the CeO_2_ interacted strongly with PU groups due to proper mixing in the polymerization method.

All of the formulated coatings were considered for outdoor exposure applications. Any coating should be considered for exposed conditions with sufficient hydrophobicity. Otherwise, moisture, air, and water can easily pass through the coating to delaminate it, and it can ultimately corrode. The most widely used method to evaluate coating hydrophobicity is the water contact angle test. The higher the contact angle value, the higher the hydrophobicity of the coating. The contact angle value highly depends on the polyol’s molecular weight and contents. Polyester-based PU showed less hydrophobicity, whereas polyether-based PU showed higher hydrophobicity. All of the recorded water contact angle values for the tested coatings are summarized in Table 2. As expected, the PU coating using a higher content of lower-molecular-weight PTMG had a smaller water contact angle. The water contact angle value continuously decreased with increasing lower-molecular-weight PTMG. However, the water contact angle value significantly increased with the inclusion of PDMS, and the value continuously increased with increasing PDMS content. The water contact angle value further increased with CeO_2_ content. The polymerized coatings also showed higher values compared to the blended coatings. Coatings with well-mixed CeO2 had higher stiffness and also resisted water penetration; ultimately, the contact angle value increased. The highest water contact angle value was recorded for the PU-11-3-3-CeO_2_-P coating, which was prepared with two PTMG polyols, a PDMS-polyol, and CeO_2_. Further, polymerization was applied during CeO_2_ use.

Any material to be considered for coating applications should have a minimum adhesive strength. The adhesive strength should remain at a minimum level throughout the coating’s lifespan. PU coatings have good adhesive strength for fabrics, metals, and PVC surfaces. The adhesive strength values are summarized in Table 2. As expected, a good adhesive strength was recorded using a higher-molecular-weight PTMG. The adhesive strength was affected by adding low-molecular-weight-based polyols. Both merits and demerits in terms of adhesive strength were found when using low-molecular-weight-based polyols. When half of the high-molecular-weight polyol was replaced with low-molecular-weight polyol (9.05 mol%), the adhesive strength decreased significantly (PU-9 coatings). Using a huge amount (PU-9 coatings) of low-molecular-weight polyols resulted in less mechanical strength, which decreased the adhesive strength. However, adhesive strength increased with the addition of 2.25, 4.525, and 6.775 mol% low-molecular-weight PDMS-polyols (PU-11, PU-13, and PU-15 coatings). The continuous increase of low-molecular-weight PDMS-polyol content in the PU-11, PU-13, and PU-15 coatings slightly affected the hardness, which was maximal in the PU-11 coatings. Eventually, adhesive strength reached a maximum at 6.775 mol% low-molecular-weight polyols. The adhesive strength increased by almost 20%–22% when adding 6.775 mol% low-molecular-weight mixed polyols (PU-11-3-3 coating). In all cases in the PU-11, PU-13 and PU-15 coatings, the adhesive strength was slightly higher when using low-molecular-weight mixed polyols, especially when compared to a single low-molecular-weight polyol. The adhesive strength value also increased with the addition of CeO_2_. The CeO_2_ acted as a filler in the coating, which was why the adhesive strength increased. The adhesive strength increased by almost 28%–40% with the addition of 1.0 wt% CeO_2_ content. At the same time, the adhesive strength values for CeO_2_-based coatings were always higher for polymerized than blended coatings. A better interaction between the CeO_2_ and polymer functional groups in the PU–CeO_2_ polymerized coating provided an extra improvement in adhesive strength. Among all coatings, the maximum adhesive strength was recorded for the PU-11-3-3-CeO_2_-P coating. This enhanced adhesive strength was due to using a proper content of mixed polyols, as well as the proper mixing of CeO_2_ during polymerization.

**Figure 3:**
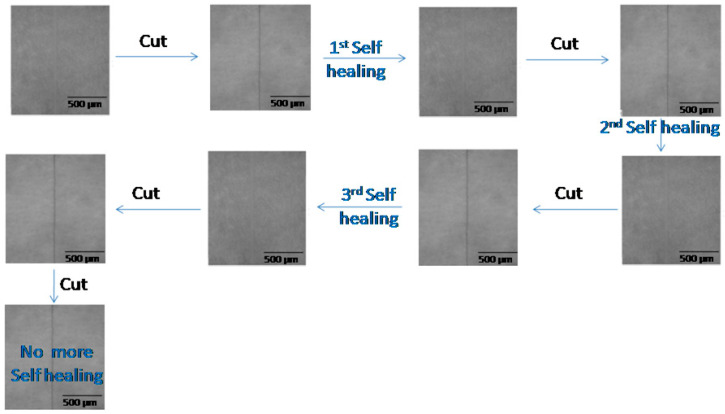
Self-healing of PU-11-3-3 coating.

The coatings were tested for self-healing via optical microscopy. All coatings showed self-healing characteristics, as all scratches healed within 12 h. However, the PU-9 coatings healed faster (9 h) than the other coatings. The fastest healed time was found to be 7 h for the PU-9-9-0 coating. As all coatings showed self-healing characteristics the first time, they were also considered for healing a second time. This time, the PU-18, PU-13, and PU-15 coatings failed to heal during the defined duration. The scratches in the PU-18, PU-13, and PU-15 coatings remained after that defined (12 h) duration. The PU-18, PU-13, and PU-15 coatings were comparatively rigid in nature. The rigid structure opposed chain mobility, and ultimately, the coating did not gain a self-healing nature. Interestingly, the PU-9 and PU-11 coatings showed self-healing characteristics the second time as the scratch disappeared within 12 h. In the PU-9 and PU-11 coatings, the flexibility was sufficient to regain the shape, as the scratch healed within that time. The proper balance of polyols made it easy to switch the polymer chain to repair the damage. The PU-9 and PU-11 coatings were also considered for a third healing test. The typical optical photographs are shown in Figure 3. These coatings showed a repairing tendency again and healed within 12 h. However, proper healing was not seen for a fourth time with the defined duration. When the coatings were scratched three times, the polymer chains lost their chain entanglement capacity. 

However, although the PU-9 coatings performed positively in terms of self-healing characteristics, they also showed very poor adhesive strength. Thus, the PU-9 coatings were not considered for the UV shielding test. The PU-11-3-3 coating showed excellent water resistance, adhesive strength, and self-healing, and this coating was considered for the UV shielding test. To minimize the number of testing coating samples, only the PU-11-3-3 coating, which showed the highest adhesive strength, was considered for mixing with CeO_2_. As mentioned before, blending and in situ polymerization were used to include the CeO_2_ content (see Table 1). These coatings were also considered for the multi-time self-healing tests within the same duration of 12 h. The in situ polymerized coating healed three times within that duration (exactly 11 h). However, the blended coating only healed the first time. Properly interacted CeO_2_ did not interfere during healing, and the healing was faster. The blended CeO_2_ had less interaction with PU groups, and free CeO_2_ may oppose polymer chain entanglement; ultimately, the healing stopped or slowed down.

**Figure 4:**
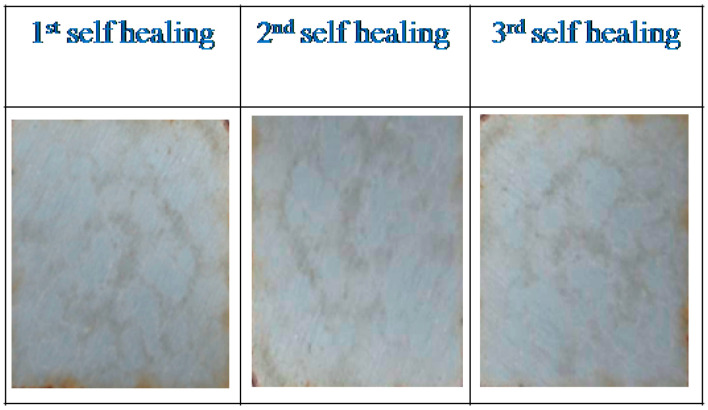
Typical photographs of exposed PU-11-3-3-CeO_2_-P-Healed coating.

**Figure 5:**
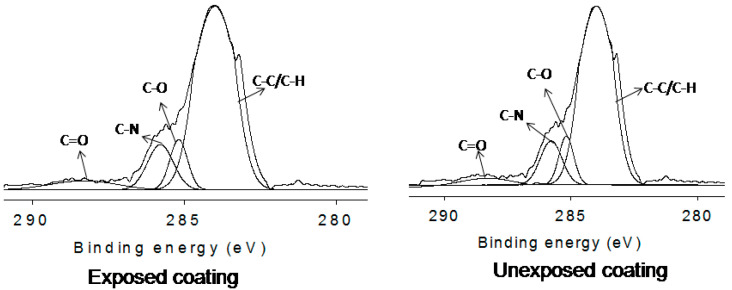
Typical deconvoluted XPS scan of coating.

To evaluate the UV shielding resistance, only five coatings (i.e., PU-11-3-3, PU-11-3-3-Ce-P, 11-3-3-Ce-P-1st-Healed, 11-3-3-Ce-P-2nd-Healed, and 11-3-3-Ce-P-3rd-Healed) were considered. Visually, it was not possible to verify the coatings’ resistance against UV degradation in the very early stages. When a coating rapidly degraded, which thus initiated metal corrosion, the degradation impact was indicated by color change, pit appearance, and delaminating. The PU-11-3-3 coating was without CeO_2_ and was completely degraded within the mentioned duration (3 months), wherein the specimen hugely corroded. The coating color changed to yellowish. Some part of the coating was also delaminated. This implies that without a UV shielding material, the coatings could not be used for UV protection. The other coatings were almost free of discoloration, brittleness, delamination, and corrosion. Typical photos of healed exposed coatings are shown in Figure 4. Visual inspection also confirmed that the PU coating using CeO_2_ had good UV shielding capacity. No significant difference was seen for these coatings. All coatings were considered for further XPS analysis. Almost the same peak appeared at the same position, confirming that they were least affected under exposure conditions. A typical deconvulated curve analysis was also done for a specific C1s area. It has been reported before that a peak appears at around 289 eV for the CO group due to chain scission [4]. As the carboxyl group also has a peak at the same position, the intensity of that peak usually increases if there is any chain scission. We found that all coatings had very slightly higher intensity peaks at that position (see Figure 5), which implies almost no degradation for all coatings. All healed coatings had similar UV shielding, as the peaks were of similar types. Although the PU-CeO_2_ coating showed promising results in self-healing and UV-shielding, the operational temperature for self-healing is quite high. The self-healing time is also longer. Above 1.0 wt% CeO_2_ content also can affect the self-healing and UV-shielding properties. Currently, our group is working to solve these issues.

## 4. Conclusions

A coating with multi-time self-healing and UV-shielding properties (i.e., PU-11-3-3-Ce-P) was prepared using a proper combination of polyols and CeO_2_ content, as well as an in situ polymerization application. The UV degradation resistance was improved by the addition of a small CeO_2_ content (1.0 wt%) without sacrificing the basic protective properties of hydrophobicity and adhesive strength. A small content (6.775 mol%) of mixed low-molecular-weight polyols contributed to 3 times better self-healing. The healed coating also showed excellent UV degradation resistance for open atmospheric conditions. This type of coating can be useful for outdoor applications.

## Figures and Tables

**Table 1:** Sample designation and composition of coatings. PDMS: polydimethyl siloxane, PTMG: poly(tetramethylene oxide)glycol.

Coating	Composition (Mol)							Polyol (mol%)			CeO_2_
	Polyol			DMPA	TEA	EDA	IPDI	PTMG (*M_n_*)		PDMS (*M_n_*)	(wt%)
	PTMG (*M_n_*)		PDMS (*M_n_*)								
	2000	650	550					2000	650	550	
PU-18	0.724	-	-	0.925	0.925	0.350	2.00	18.1	-	-	-
PU-9-9-0	0.362	0.362	-	0.925	0.925	0.350	2.00	9.05	9.05	-	-
PU-9-6-2	0.362	0.271	0.091	0.925	0.925	0.350	2.00	9.05	6.775	2.275	-
PU-9-4-4	0.362	0.181	0.181	0.925	0.925	0.350	2.00	9.05	4.525	4.525	-
PU-9-2-6	0.362	0.091	0.271	0.925	0.925	0.350	2.00	9.05	2.275	6.775	-
PU-9-0-9	0.362	-	0.362	0.925	0.925	0.350	2.00	9.05	-	9.05	-
PU-11-6-0	0.453	0.271	-	0.925	0.925	0.350	2.00	11.325	6.775	-	-
PU-11-3-3	0.453	0.1355	0.1355	0.925	0.925	0.350	2.00	11.325	3.3875	3.3875	-
PU-11-0-6	0.453	-	0.271	0.925	0.925	0.350	2.00	11.325	-	6.775	-
PU-13-4-0	0.543	0.181	-	0.925	0.925	0.350	2.00	13.575	4.525	-	-
PU-13-2-2	0.543	0.0905	0.0905	0.925	0.925	0.350	2.00	13.575	2.2625	2.2625	-
PU-13-0-4	0.543	-	0.181	0.925	0.925	0.350	2.00	13.575	-	4.525	-
PU-15-2-0	0.634	0.09	-	0.925	0.925	0.350	2.00	15.85	2.25	-	-
PU-15-1-1	0.634	0.045	0.045	0.925	0.925	0.350	2.00	15.85	1.125	1.125	-
PU-15-0-2	0.634	-	0.09	0.925	0.925	0.350	2.00	15.85	-	2.25	-
PU-11-3-3-CeO_2_-B	0.453	0.1355	0.1355	0.925	0.925	0.350	2.000	11.325	3.3875	3.3875	1.00
PU-11-3-3-CeO_2_-P	0.453	0.1355	0.1355	0.925	0.925	0.350	2.000	11.325	3.3875	3.3875	1.00

**Table 2:** Protective properties of coatings.

Coating	Hardness (Shore A)			Water Contact Angle (°)			Adhesive Strength (kgf/cm)		
PU-18	70	±	1	67	±	1	3.0	±	0.2
PU-9-9-0	53	±	1	56	±	1	1.7	±	0.3
PU-9-6-2	55	±	1	59	±	1	2.0	±	0.1
PU-9-4-4	58	±	1	63	±	1	2.2	±	0.1
PU-9-2-6	64	±	1	69	±	1	2.3	±	0.1
PU-9-0-9	69	±	1	75	±	2	2.5	±	0.1
PU-11-6-0	58	±	1	57	±	1	2.4	±	0.2
PU-11-3-3	65	±	1	70	±	1	3.6	±	0.1
PU-11-0-6	68	±	1	74	±	1	3.5	±	0.1
PU-13-4-0	61	±	1	59	±	1	2.8	±	0.1
PU-13-2-2	69	±	1	71	±	1	3.2	±	0.1
PU-13-0-4	69	±	1	71	±	1	3.2	±	0.1
PU-15-2-0	65	±	1	63	±	1	2.9	±	0.1
PU-15-1-1	68	±	1	71	±	1	3.1	±	0.2
PU-15-0-2	69	±	1	68	±	1	3.1	±	0.1
PU-11-3-3-CeO_2_-B	69	±	1	74	±	1	3.8	±	0.1
PU-11-3-3-CeO_2_-P	72	±	1	77	±	1	4.2	±	0.1
